# Massage of Bladder Meridian Relieved Anxiety Induced by Chronic Stress in Rats

**DOI:** 10.1155/2022/5639716

**Published:** 2022-12-08

**Authors:** Ping Lu, Min Fang, Lei Yao, Nan Zhang, Ke Xu, Pei He

**Affiliations:** College of Acupuncture and Massage, Shanghai University of Chinese Medicine, China

## Abstract

The aim of this paper was to explore the mechanism of bladder meridian massage (BMM) on anxiety in rats with chronic stress. Chronic stress induced rats to establish rat anxiety model. The sugar water preference (SPF), tail suspension time (TST), and forced swimming time (FST) of rats were measured. The levels of superoxide dismutase (SOD), malondialdehyde (MDA), and inflammatory cytokines in serum and hippocampus of rats were detected. Brain neurotransmitters (dopamine (DA), 5- hydroxytryptamine (5-HT), and norepinephrine (NE)) were detected by enzyme-linked immunosorbent assay (ELISA) kits. Immunohistochemistry and western blotting were used to detect autophagy protein expression in hippocampus of rats. BMM significantly increased SPF, decreased TST and FST, increased SOD level in serum and hippocampus, and decreased MDA level and cytokine level. BMM reversed the changes of neurotransmitters. At the same time, BMM significantly decreased autophagy protein expression in hippocampus of rats. The above results show that BMM significantly relieve anxiety induced by chronic stress in rats.

## 1. Introduction

With the continuous development of social economy, the accelerated pace of life, and the increasingly fierce social competition, the incidence of anxiety is increasing year by year. Anxiety disorder is a kind of mental illness characterized by emotional disorder, which is usually manifested as depression, slow thinking, exercise inhibition, appetite and sleep disorder, pessimism and world weariness, and reduced speech and movement. In severe cases, even extreme self-harm such as suicide may occur. Anxiety disorder mainly has the following clinical manifestations: depression, slow thinking, and cognitive dysfunction will activity decline. The idea or behavior of passive suicide: physical fatigue, fatigue, loss of appetite, weight loss, and so on [1, 2]. At present, anxiety has become the fourth largest disease in the world. According to the prediction of the World Health Organization (WHO), it is predicted that by 2020, the global incidence of anxiety disorder will rank second only to coronary heart disease. Due to the limitation of educational level, the influence of traditional ideas, and the diversification of anxiety patients, not only the misdiagnosis rate is high, but also the patients are repeatedly examined, resulting in great waste [3–5]. The pathogenesis of anxiety disorder is very complex and unknown.

Autophagy widely exists in eukaryotic cells and is a lysosomal-dependent degradation pathway regulated by specific genes. Under metabolic stress, such as starvation and hypoxia, cells can transport damaged, deformed and aging organelles and protein to lysosomes for digestion and degradation by autophagy [6, 7]. At the same time, the final products of autophagy, such as amino acids, fatty acids, nucleotides, and other small molecules, are rereleased into cytoplasm for cell reuse. Autophagy can be divided into three types: macrophagia, microphagia, and autophagy mediated by molecular chaperone, due to the different ways in which cytoplasmic substances are transported to lysosomes. Autophagy is usually referred to as autophagy. Autophagy formation is a multistep dynamic process, including five stages: induction, nucleation, extension, autophagy formation, and autophagy lysosome formation [8]. Therefore, autophagy plays an important role in maintaining cell homeostasis and promoting cell survival.

Traditional Chinese Medicine has shown remarkable advantages in treating anxiety disorder, especially acupuncture and massage. According to clinical reports, acupuncture and massage can effectively alleviate anxiety, but there are few basic studies. In this paper, chronic stress is used to establish rat anxiety model and evaluate the mechanism of bladder meridian massage.

## 2. Materials and Methods

### 2.1. Reagents

MDA and SOD detection kits are provided by Nanjing Jiancheng Biology Co., Ltd. (Nanjing, China). Enzyme-linked immunosorbent assay kit (ELISA) kits for interleukin (IL)-6, IL-1*β*, tumor necrosis factor (TNF)-*α*, DA, 5-HT, and NE were purchased from Nanjing Kechuang Biotechnology Co., Ltd (Nanjing, China). All antibodies were purchased from Cell Signaling Technology.

### 2.2. Animal

Thirty female SD rats (180-220 g, 6-8 weeks) were collected from Shanghai slack Experimental Animal Co., Ltd. Before the experiment, the rats were kept in SPF animal house for 3 days, and they could drink and eat freely. All experimental procedures were conducted according to Shanghai University of Chinese Medicine.

### 2.3. Experimental Process

**Figure 1:**
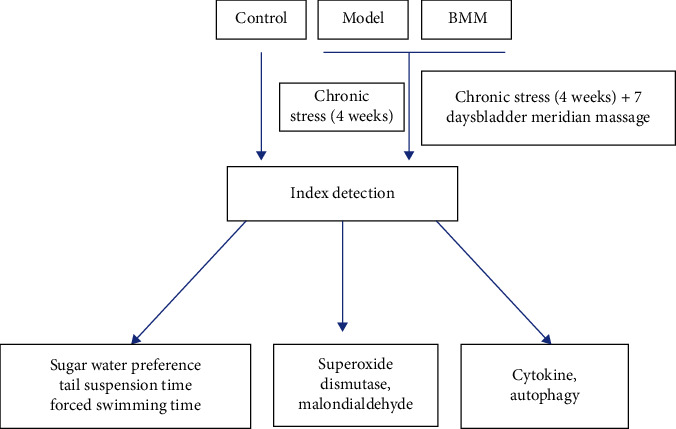
Experimental flow chart.

Thirty rats were randomly divided into control group (*n* = 10), model group (*n* = 10), and BMM group (*n* = 10). Except the control group rats, the other rats were exposed to chronic stress (the rats in each group were bound with a binding frame. They were bound at 18 : 00-6 : 00 every day for 3 hours, and the binding time point was random) for 4 weeks [9], and then intervened. In the BMM group, the rats were fixed with a self-made device “quantitative tester of pressing technique”, and quantitative pressing stimulation. The self-developed “YLS-34A rat acupoint pressing stimulator” was used to fix the rats, and quantitative pressing stimulation was given to the bladder meridian on both sides of the spinal column of the rats. Once a day for 7 consecutive days. 250 g force was set for intervention and was given to the bladder meridians on both sides of the spine of the rats for 30 min. Stimulate once a day and intervene continuously for 7 days. 24 h after the end of the last intervention, the anxiety behaviors of rats in different treatment groups were tested by the above anxiety behavior test indicators and other tests; the experimental flow is shown in Figure 1.

### 2.4. Behavioral Test

#### 2.4.1. Sucrose Preference Test (SPF)

SPF experiment is a classic experiment to detect the typical symptoms of anxiety disorder or depression. Three days before the experiment, the SD rats were preacclimatized: 1% sucrose water was placed on the cage, and the next day it was changed into ordinary drinking water to make the SD rats adapt to the taste of sugar water. Before the formal test, SD rats were fasted and watered for 24 h and then given 1 bottle of 1% sugar water and 1 bottle of ordinary drinking water at the same time. The drinking time was 1 h. Then, the consumption of two bottles of liquid was observed and recorded, and the preference percentage of sugar water was calculated. The calculation formula was as follows:sugar water consumption rate = [sugar water consumption/(sugar water consumption + water consumption)] × 100%.

#### 2.4.2. Tail Suspension Test (TST)

In this experiment, within 24 hours after the last compression treatment, rats were isolated from hearing and vision in a quiet environment. Stick the tape on the tail of the rats (about 50 cm from the ground) and hang the rats alone. Each rat was suspended for 6 minutes. The rest time in the last 4 minutes were recorded.

#### 2.4.3. Forced Swimming Test (FST)

In this experiment, within 24 hours after the last compression treatment, the FST was performed as previously described. In short, rats were placed in a glass cylinder (37 cm in height and 34 cm in diameter) filled with water, and the height of water could reach 10 cm (25°C). Static time was recorded as the time when rats float in the water, and only done small movements needed to keep their heads off the water. The total duration of immobility was measured in the last 4 minutes of the 6-minute test period.

### 2.5. Detection of DA, NE, and 5-HT in Hippocampus

The levels of DA, NE, and 5-HT such as IL-6, IL-1*β*, and TNF-*α* in serum and hippocampus were determined by ELISA kits according to the manufacturer's instructions.

### 2.6. Detection of Inflammatory Cytokines in Serum and Hippocampus

The levels of inflammatory cytokines such as IL-6, IL-1*β*, and TNF-*α* in serum and hippocampus were determined by ELISA kits according to the manufacturer's instructions.

### 2.7. Detection of SOD and MDA in Serum and Hippocampus

Commercial reagents were used to detect SOD and MDA in serum and hippocampus of rats, and the instructions of the kit were strictly followed.

### 2.8. Immunohistochemistry

Immunohistochemical staining was used to further detect the expression of LC-32 in hippocampus. In short, the coronal section was cut horizontally in the dorsal hippocampus and processed, and the paraffin section of hippocampus was dewaxed, rehydrated, and incubated in 3% hydrogen peroxide. Samples were blocked with 3% bovine serum albumin and incubated with primary antibody at 4°C overnight. Secondary antibody and tertiary antibody were incubated at 37°C for 20 minutes, respectively. Then, the samples were stained with DAB and restained with hematoxylin. After dehydration and drying, the slices were fixed and observed under a microscope.

### 2.9. Western Blot Analysis

100 mg hippocampal tissue was homogenized in ice-cold RIPA buffer (0.1% phenylmethanesulfonyl fluoride). The solution was centrifuged, and the protein concentration of supernatant was determined by BCA kit. Samples were loaded by SDS-polyacrylamide gel electrophoresis, and the separated protein was transferred to polyvinylidene fluoride membrane. After sealing with 5% skim milk, polyvinylidene fluoride membrane was incubated with primary antibody at 4°C overnight. After washing with Tris-buffered saline-Tween 20 (TBST), the polyvinylidene fluoride membrane was incubated with the second antibody at room temperature. These bands are visualized by bioelectrochemical XRS system. ImageJ uses image analysis software for density analysis.

### 2.10. Statistical Analysis

All data are expressed in average standard deviation and were analyzed by ANOVA, and the back testing of Tukey was performed by SPSS 17.0. *P* value less than 0.05 is considered as significant difference.

## 3. Results

### 3.1. Effect of BMM on SOD and MDA in Serum of Anxiety Rats

**Figure 2:**
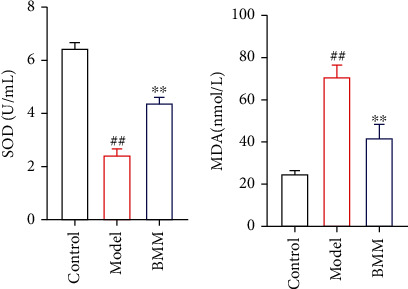
Effect of BMM on SOD and MDA in serum of anxiety rats. Values are expressed as means ± SDs. Compared with control: ^##^*P* < 0.01; compared with model: ^∗^*P* < 0.05, ^∗∗^*P* < 0.01.

Compared with control rats, serum SOD in model rats significantly decreased, and MDA significantly increased. Compared with model rats, BMM significantly decreased serum MDA and increased serum SOD level. The results are shown in Figure 2.

### 3.2. Effect of BMM on SOD and MDA in Hippocampus of Anxiety Rats

**Figure 3:**
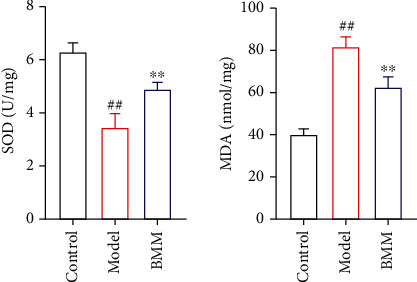
Effect of BMM on SOD and MDA in hippocampus of anxiety rats. Values are expressed as means ± SDs. Compared with control: ^##^*P* < 0.01; compared with model: ^∗^*P* < 0.05, ^∗∗^*P* < 0.01.

Compared with control rats, serum SOD significantly decreased and MDA significantly increased in hippocampus of model rats. Compared with model rats, BMM significantly decreased serum MDA and increased serum SOD level. The results are shown in Figure 3.

### 3.3. Effects of BMM on IL-6, IL-1*β ,*and TNF-*α* in Hippocampus of Anxiety Rats

**Figure 4:**
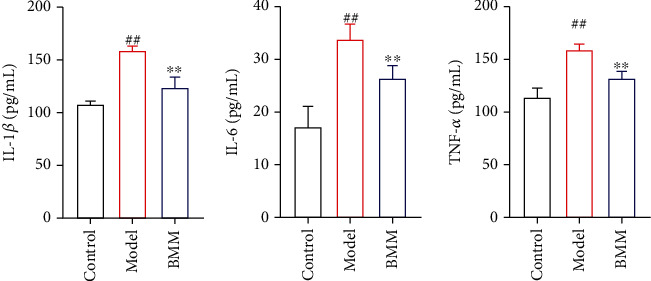
Effects of BMM on IL-6, IL-1*β*, and TNF-*α* in hippocampus of anxiety rats. Values are expressed as means ± SDs. Compared with control: ^##^*P* < 0.01; compared with model: ^∗^*P* < 0.05, ^∗∗^*P* < 0.01.

Compared with control rats, IL-6, IL-1*β*, and TNF-*α* in serum of model rats were significantly increased, and BMM significantly decreased serum IL-6, IL-1*β*, and TNF-*α* levels compared with model rats (Figure 4).

### 3.4. Effects of BMM on IL-6, IL-1*β*, and TNF-*α* in Serum of Anxiety Rats

**Figure 5:**
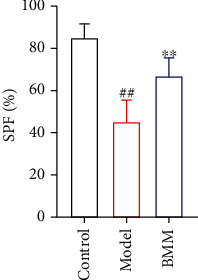
Effects of BMM on IL-6, IL-1*β*, and TNF-*α* in serum of anxiety rats. Values are expressed as means ± SDs. Compared with control: ^##^*P* < 0.01; compared with model: ^∗^*P* < 0.05, ^∗∗^*P* < 0.01.

Compared with control rats, IL-6, IL-1*β*, and TNF-*α* in hippocampus of model rats were significantly increased, and BMM significantly decreased serum IL-6, IL-1*β*, and TNF-*α* levels in hippocampus compared with model rats (Figure 5).

### 3.5. Effect of BMM on SPF in Anxiety Rats

**Figure 6:**
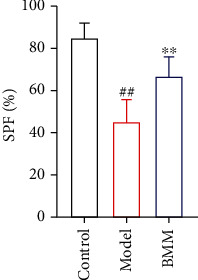
Effect of BMM on SPF in anxiety rats. Values are expressed as means ± SDs. Compared with control: ^##^*P* < 0.01; compared with model: ^∗^*P* < 0.05, ^∗∗^*P* < 0.01.

Compared with control rats, SPF of model rats significantly decreased, and BMM significantly increased SPF level compared with model rats (Figure 6).

### 3.6. Effect of BMM on FST and TST in Anxiety Rats

**Figure 7:**
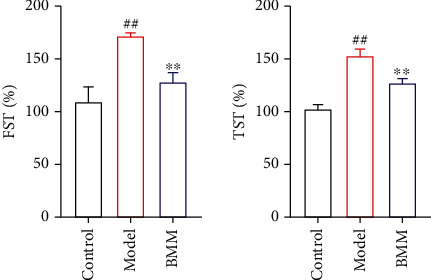
Effect of BMM on FST and TST in anxiety rats. Values are expressed as means ± SDs. Compared with control: ^##^*P* < 0.01; compared with model: ^∗^*P* < 0.05, ^∗∗^*P* < 0.01.

Compared with control rats, TST and FST in model rats significantly increased, and BMM significantly decreased TST and FST levels compared with model rats (Figure 7).

### 3.7. Effect of BMM on DA, NE, and 5-HT in Hippocampus

**Figure 8:**
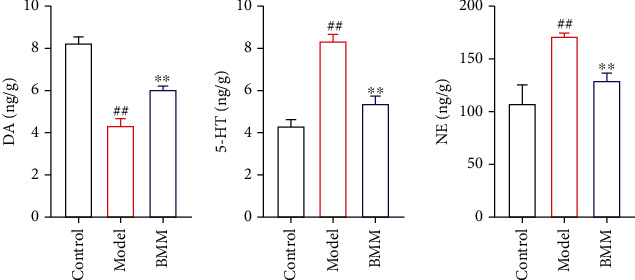
Effect of BMM on DA, NE and 5-HT in hippocampus. Values are expressed as means ± SDs. Compared with control: ^##^*P* < 0.01; compared with model: ^∗^*P* < 0.05, ^∗∗^*P* < 0.01.

Compared with the control group, DA was significantly decreased; 5-HT and NE were significantly increased in the model group, while DA was significantly increased; 5-HT and NE were significantly decreased in the BMM group compared with the model group (Figure 8).

### 3.8. Effect of BMM on LC-32 in Hippocampus of Anxiety Rats by Immunohistochemistry

**Figure 9:**
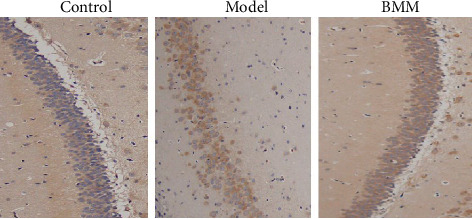
Effect of BMM on LC-32 in hippocampus of anxiety rats by immunohistochemistry.

Compared with control group, LC-32 was significantly increased in model group, and BMM significantly decreased LC-32 level in hippocampus compared with model group rats (Figure 9).

### 3.9. Effect of BMM on LC-32 in Hippocampus of Anxiety Rats by Western Blot

**Figure 10:**
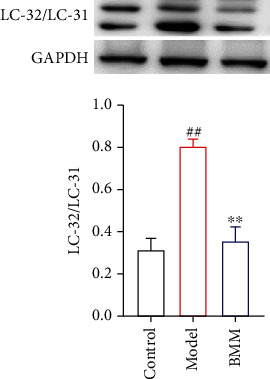
Effect of BMM on LC-32 in hippocampus of anxiety rats by western blot. Values are expressed as means ± SDs. Compared with control: ^##^*P* < 0.01; compared with model: ^∗^*P* < 0.05, ^∗∗^*P* < 0.01.

Compared with control group, LC-32 was significantly increased in model group, and BMM significantly decreased LC-32 level in hippocampus compared with model group rats (Figure 10).

## 4. Discussion

The purpose of this study was to explore the antianxiety effect of BMM on anxiety rats and its possible mechanism. The results showed that BMM can significantly reduce anxiety behavior of anxiety rats.

The occurrence of anxiety disorder is related to complex multifactorial etiology [10]. As we all know, anxiety patients show high levels of proinflammatory cytokines [11]. The current research shows that BMM has effects on the behavior of rats induced by chronic stress. The antianxiety activity of BMM was closely related to its anti-inflammatory effects [12]. More and more evidences shown that stress, especially chronic stress, played a key role in the pathophysiology of anxiety. By randomly assigning different low-intensity stress factors to simulate human anxiety state and stimulating animals with chronic low-intensity stress, the anxiety model of chronic stress wood was established. This model was similar to the clinical symptoms of patients with anxiety disorder and was widely used to screen drugs with antianxiety activity [13]. Behavioral experiments play an important role in evaluating whether drugs have antianxiety activity. Current research shown that the rats in the model group obviously reduced their SPF. SPF is a classic experiment to detect the typical symptoms of anxiety disorder or depression [14]. The results of this study showed that BMM significantly increased SPF in anxiety rats. OFT, FST, and TST are commonly used methods to evaluate anxiety disorder in experiments, because they have high predictive validity [15]. In the present study, BMM improved the behavior of anxiety rats. Behavioral tests included FST and TST tests, and the results showed that BMM significantly decreased FST and TST levels.

According to cytokine hypothesis, psychological stress and internal stress may cause anxiety disorder through inflammatory process [16]. The proinflammatory cytokine IL-1 has been found to affect neuronal plasticity, which is the basis of learning and memory, while cognitive ability is often impaired in severe depression. In addition, it is reported that interleukin-6 is also related to synaptic plasticity, neuromodulation, and neurogenesis [17]. Tumor necrosis factor-*α* (TNF-*α*) has been widely used as a biomarker of stress, which plays a major role in controlling innate immunity of brain in serum and hippocampus. The results showed that the levels of IL-6, IL-1*β*, and TNF-*α* in serum and hippocampus of rats in BMM were significantly lower than those in model group.

Autophagy widely exists in eukaryotic cells and is a lysosomal-dependent degradation pathway regulated by specific genes. Under metabolic stress such as starvation and hypoxia, cells can transport damaged, deformed and aging organelles and protein to lysosomes for digestion and degradation by autophagy. Autophagy of hippocampal neurons in anxiety rats was significantly activated; autophagy increased, and LC-32/LC-31 ratio increased. Consistent with the findings of this experiment, the results of immunohistochemistry and western blot showed that LC-32/LC-31 in hippocampus of rats in model group significantly increased, while LC-32/LC-31 in hippocampus of rats in BMM group significantly decreased.

In a word, BMM significantly improved anxiety induced by chronic stress in rats, and its mechanism is related to the regulation of hippocampal autophagy.

## Data Availability

The data used to support the findings of this study are available from the corresponding authors upon request.

## Abbreviations

BMM: Bladder meridian massage
SPF: Sugar water preference
TST: Tail suspension time
FST: Forced swimming time
SOD: Superoxide dismutase
MDA: Malondialdehyde
DA: Dopamine
5-HT: 5-hydroxytryptamine
NE: Norepinephrine
ELISA: Enzyme linked immunosorbent assay
WHO: World Health Organization
IL: Interleukin
TNF: Tumor necrosis factor.

## References

[1] Sawamura J., Morishita S., Ishigooka J. (2016). Symmetrical treatment of diagnostic and statistical manual of mental disorders, fifth edition, for major depressive disorders. *Source Code for Biology and Medicine*.

[2] Murray C. J., Vos T., Lozano R. (2012). Disability-adjusted life years (DALYs) for 291 diseases and injuries in 21 regions, 1990-2010: a systematic analysis for the Global Burden of Disease study 2010. *Lancet*.

[3] Organization W. H. (2008). *The Global Burden of Disease 2004 Update*.

[4] Gu L., Xie J., Long J. (2013). Epidemiology of major depressive disorder in mainland china: a systematic review. *PLoS One*.

[5] Kessler R. C., Berglund P., Demler O. (2003). The epidemiology of major depressive disorder: results from the National Comorbidity Survey Replication (NCS-R). *Jama*.

[6] Yang Z., Klionsky D. J. (2010). Eaten alive: a history of macroautophagy. *Nature Cell Biology*.

[7] Levy J. M., Thorburn A. (2011). Targeting autophagy during cancer therapy to improve clinical outcomes. *Pharmacology & Therapeutics*.

[8] Choi A. M., Ryter S. W., Levine B. (2013). Autophagy in human health and disease. *The New England Journal of Medicine*.

[9] Tang M., Huang H., Li S. (2019). Hippocampal proteomic changes of susceptibility and resilience to depression or anxiety in a rat model of chronic mild stress. *Translational Psychiatry*.

[10] Feng D.-d., Tang T., Lin X.-p. (2016). Nine traditional Chinese herbal formulas for the treatment of depression: an ethnopharmacology, phytochemistry, and pharmacology review. *Neuropsychiatric Disease and Treatment*.

[11] Luo D. D., An S. C., Zhang X. (2008). Involvement of hippocampal serotonin and neuropeptide Y in depression induced by chronic unpredicted mild stress. *Brain Research Bulletin*.

[12] Rodrigues A. L. S., da Silva G. L., Mateussi A. S. (2002). Involvement of monoaminergic system in the antidepressant-like effect of the hydroalcoholic extract of _Siphocampylus verticillatus_. *Life Sciences*.

[13] Han C., Yeh T. L., Kato M., Sato S., Chang C. M., Pae C. U. (2013). Management of chronic depressive patients with residual symptoms. *CNS Drugs*.

[14] Kim S. W., Kang H. J., Bae K. Y. (2017). Interactions between pro-inflammatory cytokines and statins on depression in patients with acute coronary syndrome. *Progress in Neuro-Psychopharmacology & Biological Psychiatry*.

[15] Antunes M. S., Jesse C. R., Ruff J. R. (2016). Hesperidin reverses cognitive and depressive disturbances induced by olfactory bulbectomy in mice by modulating hippocampal neurotrophins and cytokine levels and acetylcholinesterase activity. *European Journal of Pharmacology*.

[16] Yang D., Li Q., Fang L. (2011). Reduced neurogenesis and pre-synaptic dysfunction in the olfactory bulb of a rat model of depression. *Neuroscience*.

[17] Masi G., Brovedani P. (2011). The hippocampus, neurotrophic factors and depression. *CNS Drugs*.

